# Effects of concentrate input on nutrient utilization and methane emissions of two breeds of ewe lambs fed fresh ryegrass

**DOI:** 10.1093/tas/txy106

**Published:** 2018-10-11

**Authors:** Chunmei Wang, Yiguang Zhao, Aurélie Aubry, Gareth Arnott, Fujiang Hou, Tianhai Yan

**Affiliations:** 1 Agri-Food and Biosciences Institute, Hillsborough, Co Down, UK; 2 State Key Laboratory of Grassland Agro-Ecosystems, Key Laboratory of Grassland Livestock Industry Innovation, Ministry of Agriculture and Rural Affairs, College of Pastoral Agriculture Science and Technology, Lanzhou University, Lanzhou, Gansu, China; 3 Institute for Global Food Security, School of Biological Science, Queen’s University Belfast, Belfast, UK

**Keywords:** concentrate supplement, ewe lamb, fresh grass, methane emission, nutrient utilization

## Abstract

The objective of this study was to evaluate if high-quality grass could sustain a similar feeding efficiency to concentrate meals for two breeds of lowland ewe lambs. Sixteen lowland ewe lambs of approximately 13 mo age and 61.5 ± 5.28 kg live weight were used in a 2 × 2 factorial study, with 2 diets (fresh perennial ryegrass [*Lolium perenne*] vs. fresh perennial ryegrass plus 0.5 kg/d fresh concentrate) × 2 breeds (Highlander vs. Texel). Grass was cut daily in the morning from a single zero-grazing sward and offered ad libitum. The animals were individually housed in pens and fed experimental diets for an adaptation phase of 19 d, and then transferred to respiration calorimeter chambers, remaining there for 5 d, with feed intake, feces and urine outputs, and methane (CH_4_) emissions measured during the final 4 d. There were no significant interaction effects between diets and breeds on any variables. Ewe lambs offered 0.5 kg/d concentrate supplementation had slightly greater DM intake and energy (GE, DE, and ME) intake, but had significantly higher N intake and N excretion in feces and urine than those fed the grass-only diet. However, diets had no significant effects on nutrient digestibility, energy or N utilization, or CH_4_ emission. Texel breed had a significantly lower DM intake and CH_4_ emissions per kg live weight, whereas the breed had no significant effect on nutrient digestibility or energy or N utilization. These results implicate that good quality grass could sustain high nutrient utilization efficiency as effectively as diets supplemented with concentrates for ewe lamb production. The two breeds of lowland ewe lambs can utilize good quality grass at a similar level of efficiency.

## INTRODUCTION

Grass is the most important feed source for ruminant livestock. Through grazing and conservation, grass can provide energy and nutrients to meet over 50% requirements in cattle and sheep production (Fanchone et al., 2013). For example, in Northern Ireland, there is a focus on pasture-based sheep production, as the prevailing climate conditions enable grass pasture offering feed to sheep all year round. However, there are a range of challenges in grass-based system including the large variations in grass availability and grass feeding values influenced by growing season and the stage of growth. As such, sheep/cattle grazing in the poor quality swards may require concentrate supplementation to sustain the targeted production level and to minimize disadvantage impacts of fluctuation in pasture availability and/or quality (Guyader et al., 2016).

Concentrate supplementation is an effective strategy to optimize the rumen microbial activity through the balanced supply of fermentable carbohydrates and N, especially for diets containing low-quality grass. Furthermore, concentrate supplementation usually results in increased DM intake (Blaxter et al., 1961) and nutrient digestibility (Eng et al., 1964), and decreased N losses especially urinary N excretion (Hoekstra et al., 2007; Zhao et al., 2015) and CH_4_ emissions (Yan et al., 2010). However, the problem of low microbial protein yield in cattle and sheep fed poor quality forages cannot simply be solved or completely compensated by supplementing high amounts of concentrates (Pathak, 2008), which directly affects the efficiency of N utilization (Fanchone et al., 2013). It is therefore critical for the sheep production industry to explore feeding and management approaches to lower feed costs by reducing supplementary concentrates while maintaining high DM intake to meet the nutritional requirements. Some previous studies demonstrated that increasing grass quality could improve the concentrate-sparing effect (Keady and Hanrahan et al., 2015) and offset the effects of low concentrate inputs on feed intake and digestibility (Blaxter et al., 1961; Ramos et al., 2009). However, the question still remains open regarding whether good quality grass could be used to replace all concentrate supplementation without compromising feed intake, nutrient utilization, and enteric CH_4_ emissions in lowland ewe lambs. Furthermore, there is little information available on evaluation of effects of different breeds of lowland replacement ewes offered good quality fresh grass in a zero-grazing study on feed intake, energy and N utilization efficiency, and CH_4_ emissions. Hence, the objective of the present zero-grazing study was to evaluate if feeding lowland ewe lambs (Highlander vs. Texel) with good quality grass could sustain a high nutrient intake, digestibility, and N and energy utilization efficiency as effectively as using a diet supplemented with concentrate feed.

## MATERIALS AND METHODS

The experiment was conducted under the regulations of Department of Health, Social Services and Public Safety of Northern Ireland in accordance with the Animals (Scientific Procedures) Act 1986 (Home Office, 1986).

### Experimental Design and Diets

Sixteen ewe lambs at approximately 13 mo age and 61.5 ± 5.28 kg live weight were used in a 2 × 2 factorial design study, with 2 diets (fresh perennial ryegrass [Grass] vs. fresh perennial ryegrass plus 0.5 kg/d fresh concentrate [Grass + Conc.]) × 2 breeds (Highlander vs. Texel). Each treatment had four lambs and were balanced within each breed for animal age and live weight. The sheep were housed in individual pens for 19 d, then transferred to individual crates that were placed in climate-controlled open circuit respiration chambers (one sheep per crate per chamber) and remained there for 5 d with nutrient digestibility and CH_4_ emissions measured during the final 4 d. All equipment, sampling procedures, analytical methods, and calibrate of chambers were described by Zhao et al. (2015). Water was freely available during the period of study.

Grass was fed ad libitum, with feed levels designed to ensure 10% orts. Residual feeds were removed and weighed, before total daily grass allowance was offered in a single meal ad libitum in the morning (10.00 a.m.), with the concentrate portion given at the same time for the treatment receiving concentrate. Fresh grass was cut daily in the morning from a single zero-grazing sward during the first regrowth. Plots were initially trimmed throughout at a residual height of 4 cm and then allowed for regrowth for 2 to 3 wk before harvesting in simulation to grazing condition. Each plot was used for 1 wk. Sward heights were measured using a rising plate meter (Jenquip folding plate pasture meter; Jenquip, Feilding, New Zealand), with 20 sward height measurements being taken at random in a “W” shape across the area designated for harvesting. The mean aboveground herbage masses for the cutting areas were then estimated using the following linear equation: herbage mass (kg DM/ha) = (sward height (cm) × 316) + 330 (Jiao et al., 2014). The required plot size was calculated by the feed intake and grass masses. The ingredient composition of the concentrates offered (DM basis) was as follows: 33.3% barely, 25.6% beet pulp, 25.6% soybean meal, 10.3% maize meal, 3.1% Molaferm (United Molasses GB, London, United Kingdom), and 2.1% vitamin and 2.1% mineral premix (Trouw Intensive Lamb; Trouw UK, Cheshire, United Kingdom).

Grass contained, on average, 0.197 kg/kg DM, 18.7 MJ/kg DM of gross energy (GE), 0.069 ash (kg/kg DM basis), 0.150 crude protein (CP), 0.459 neutral detergent fiber (NDF), 0.236 acid detergent fiber (ADF), 0.215 water-soluble carbohydrates (WSC), and 0.036 ether extract (EE). The concentrate contained 0.878 kg/kg DM, 17.8 MJ/kg DM of GE, and 0.069, 0.206, 0.177, 0.119, and 0.023 kg/kg DM of ash, CP, NDF, ADF and EE concentrations, respectively.

### Measurements

Live weight was weighed at the beginning and at the end of adaptation and measurement periods. Feed offered and refused were recorded daily for each ewe during the experiment period. Fresh and residual forage samples were retained for determination of DM content at 85 °C for 24 h. Dried samples were bulked twice weekly, milled (0.8 mm pore size), and analyzed for ash, CP, NDF, ADF, EE and GE contents. Meanwhile, another sample of fresh herbage also was obtained twice weekly and dried at 60 °C for 24 h for determination of WSC concentration. Concentrates were sampled daily during the measurement period, bulked as a single sample and dried at 85 °C for 24 h. The dried samples were then milled (0.8 mm sieve size) for determination of ash, GE, CP, NDF, ADF, EE, and starch concentrations.

During the period of digestibility trial, feces and urine (10 mL 35% sulfuric acid added to the urine collection jar) outputs from each ewe were recorded daily. Feces and urine (as 20% proportion of total urine output) samples were stored in 4 °C during the first 3 d. After the last day of collection, the feces and urine samples were thoroughly mixed and representative samples were taken for further analysis. Each feces sample was divided into two subsamples. One portion was used for measuring N concentration on a fresh basis and the other was dried at 100 °C for 48 h and then ground (0.8 mm sieve size) for determination of ash, GE, NDF, and ADF concentrations. The urine samples were used for the measurement of GE and N concentration with GE measured in 10 mL freeze-dried samples, which were contained in self-sealing polythene bags of known weight and energy concentration.

Dry matter concentration was determined using forced draught oven (MINI/75; Genla, Cheshire, United Kingdom). Gross energy concentrations in feed, feces, and urine samples were determined in an isoperibol bomb calorimeter (Parr Instrument Co., Moline, IL). The N concentration was analyzed on a fresh basis for feces and urine samples and on a DM basis for feed samples using a Tecator Kjeldahl Auto 1030 Analyzer (Foss Tecator AB, Höganäs, Sweden). Manure N (MN) was calculated as the sum of fecal N (FN) and urine N (UN). The concentrations of NDF (without alpha-amylase and sodium sulfite) and ADF, expressed exclusive of residual ash, were determined using the Tecator Fibertec System (Foss Tecator AB) following the procedures of Robertson and Van Soest (1981). Grass WSC concentration was analyzed using a Continuous Segmented Flow Analyzer (SEAL Analytical Ltd., Southampton, United Kingdom) and the method of McDonald and Henderson (1964). Ash was measured by combustion using a muffle furnace (Vecstar Ltd., Chesterfield, United Kingdom) at 550 °C for 10 h (method 942.05; AOAC, 1990). Lipid concentration was measured using Foss Soxtec 2043 Fat Extraction System (Foss Tecator AB).

### Statistical Analysis

Data were analyzed using general linear mixed models using the GenStat statistical software (16th edition). The model was y_ijk_ = μ + Diet_i_ + Breed_j_ + (Diet*Breed)_ij_ + Date_k_ + e_ijk_, where y_ijk_ was observed value, μ was overall constant, Diet_i_ was the fixed effect of diet i (i is the diet level assigned to unit ijk), Breed_j_ was the fixed effect of breed j (j is the breed assigned to unit ijk), (Diet*Breed)_ij_ was the interaction effect, Date_k_ was the random effect of date k (k was the date of animal assigned to run through open circuit respiration chamber), and e_ijk_ was the random (residual) error. Significance was at *P* ˂ 0.05 and 0.05 ≤ *P* ˂ 0.1 was declared as trend toward significance.

There were no significant interaction effects between diets and breeds on any variable for BW, feed intake, apparent digestibility, energy metabolism, enteric CH_4_ emission, and N utilization, so these interaction effects are not presented.

## RESULTS

### Nutrition Intake and Digestibility

The effects of diet and breed on BW, nutrition intake, and digestibility are presented in Table 1. Ewes supplemented fresh concentrates at 0.5 kg/d had an almost significantly higher DM intake (*P* = 0.059) and OM intake (*P* = 0.058) than those offered solely fresh grass, although feeding concentrates significantly reduced grass DM intake (*P* < 0.05). Breed had no significant effect on nutrition intake (kg/d) or digestibility, except for the intake capacity (DMI/BW) which was significantly higher (*P* < 0.05) for Highlander than that for Texel.

**Table 1.** Effects of diet and breed on feed intake and digestibility of ewe lambs (*n* = 16)

	Diet				Breed			
	Grass	Grass+ conc.	SEM	^*^ *P*	Highlander	Texel	SEM	^*^ *P*
Feed intake, kg/d								
Grass DM	1.62	1.37	0.068	0.021	1.56	1.43	0.065	0.173
Total DM	1.62	1.81	0.068	0.059	1.78	1.65	0.065	0.173
DM intake/BW, g/kg	27.7	28.1	0.82	0.723	29.2	26.6	0.78	0.039
OM	1.51	1.68	0.064	0.058	1.66	1.53	0.061	0.175
ADF	0.38	0.38	0.017	0.959	0.39	0.36	0.016	0.193
NDF	0.74	0.75	0.032	0.906	0.78	0.71	0.030	0.165
Digestibility, kg/kg or MJ/MJ								
DM	0.817	0.821	0.0091	0.664	0.822	0.816	0.0086	0.672
OM	0.833	0.838	0.0090	0.575	0.838	0.833	0.0085	0.670
Digestible OM in DM	0.776	0.779	0.0082	0.750	0.781	0.775	0.0078	0.625
N	0.703	0.693	0.0284	0.672	0.715	0.681	0.0269	0.404
GE	0.801	0.804	0.0107	0.691	0.804	0.801	0.0101	0.789
ADF	0.808	0.795	0.0119	0.700	0.806	0.797	0.0113	0.603
NDF	0.785	0.770	0.0140	0.700	0.780	0.775	0.0132	0.781

### Energy Utilization

The effects of diet and breed on energy intake and utilization are presented in Table 2. Neither concentrate supplementation nor breed had any significant effect on any variable of energy utilization, although ewes offered concentrate had slightly higher GE intake (*P* = 0.093), DE intake (*P* = 0.071), and ME intake (*P* = 0.065) than those fed solely fresh grass.

**Table 2.** Effects of diet and breed on energy utilization of ewe lambs (*n* = 16)

	Diet				Breed			
	Grass	Grass+ Conc.	SEM	^*^ *P*	Highlander	Texel	SEM	^*^ *P*
Energy intake and output, MJ/d								
GE intake	30.3	33.3	1.24	0.093	33.0	30.6	1.17	0.166
Fecal E output	6.0	6.5	0.45	0.446	6.5	6.1	0.42	0.603
Urine E output	1.0	1.1	0.06	0.834	1.0	1.0	0.06	0.630
CH_4_-E output	1.5	1.8	0.09	0.176	1.7	1.5	0.09	0.223
DE intake	24.3	26.8	0.96	0.071	26.5	24.5	0.91	0.146
ME intake	21.8	23.9	0.88	0.065	23.8	22.0	0.84	0.152
Energy utilization, MJ/MJ								
DE/GE	0.801	0.804	0.0107	0.691	0.804	0.801	0.0101	0.789
ME/GE	0.718	0.720	0.0096	0.476	0.721	0.717	0.0091	0.780
ME/DE	0.897	0.895	0.0043	0.429	0.896	0.896	0.0040	0.913

### Enteric CH_4_ Emission

The effects of diet and breed on enteric CH_4_ emissions are presented in Table 3. Either diet or breed had no significant effects on any CH_4_ emission variables, except for CH_4_ per BW, which was significantly higher (*P* < 0.05) for Highlander than that for Texel.

**Table 3.** Effects of diet and breed on enteric CH_4_ emissions of ewe lambs (*n* = 16)

	Diet				Breed			
	Grass	Grass+Conc.	SEM	^*^ *P*	Highlander	Texel	SEM	^*^ *P*
CH_4_ production, g/d	27.4	31.6	1.67	0.176	31.0	28.0	1.58	0.223
CH_4_/DM intake, g/kg	17.0	17.5	0.89	0.793	17.5	17.0	0.84	0.716
CH_4_/OM intake, g/kg	18.3	18.8	0.10	0.780	18.8	18.3	0.91	0.725
CH_4_/digestible DM intake, g/kg	20.8	21.3	1.01	0.977	21.2	20.9	0.95	0.781
CH_4_/digestible OM intake, g/kg	21.9	22.4	1.05	0.995	22.4	22.0	1.00	0.784
CH_4_/BW, g/kg	0.47	0.49	0.023	0.743	0.52	0.44	0.021	0.028
CH_4_-E/GE intake, MJ/MJ	0.0502	0.0527	0.00263	0.733	0.0522	0.0508	0.00249	0.706
CH_4_-E/DE intake, MJ/MJ	0.0628	0.0656	0.00297	0.910	0.0648	0.0636	0.00281	0.767
CH_4_-E/ME intake, MJ/MJ	0.0701	0.0736	0.00364	0.942	0.0725	0.0712	0.00344	0.798

### Nitrogen Utilization

The effects of diet and breed on N utilization of ewe lambs are presented in Table 4. Ewes offered concentrates had a higher N intake (*P* < 0.001), and higher N excretion in feces (*P* < 0.05), urine (*P* < 0.01), and manure (*P* < 0.01), but had no influences on fecal, urinary, or retained N outputs, expressed as the proportion of N intake. Breed had no significant effect on any N utilization variable.

**Table 4.** Effects of diet and breed on N utilization of ewe lambs (*n* = 16)

	Diet				Breed			
	Grass	Grass+ Conc.	SEM	^*^ *P*	Highlander	Texel	SEM	^*^ *P*
N intake/and output, g/d								
N intake	41.0	46.6	1.05	<0.001	45.0	42.5	0.99	0.110
Feces N output	10.7	13.8	0.85	0.031	12.2	12.3	0.80	0.945
Urine N output	18.7	20.6	0.80	0.002	20.2	19.1	0.76	0.333
Manure N output	29.4	34.4	1.23	0.002	32.4	31.4	1.17	0.553
Retained N	11.5	12.2	1.57	0.261	12.6	11.1	1.48	0.507
N utilization, g/g								
Fecal N/N intake	0.297	0.307	0.0284	0.672	0.285	0.319	0.0027	0.404
Urine N/N intake	0.426	0.443	0.0273	0.486	0.431	0.438	0.0258	0.857
Manure N/N intake	0.723	0.749	0.0370	0.458	0.716	0.756	0.0350	0.437
Retained N/N intake	0.277	0.251	0.0370	0.458	0.284	0.244	0.0350	0.437
Feces N/Manure N	0.407	0.407	0.0257	0.420	0.402	0.411	0.0243	0.793
Urine N/Manure N	0.593	0.593	0.0257	0.420	0.598	0.589	0.0243	0.793
Urine N/Feces N	1.71	1.48	0.1288	0.960	1.61	1.59	0.1219	0.924

## DISCUSSION

### Effects on Feed Intake and Nutrient Utilization

In this study, the concentrate substitution rate was 0.56 kg/kg. Some previous studies found that effects of concentrate supplementation on voluntary feed intake and diet digestibility were affected by the quality of forage (Keady and Hanrahan, 2015). For example, Blaxter et al. (1961) reported a linear increase in substitution rates for voluntary forage intake by concentrate input in sheep when offered good rather than poor quality hay at a concentrate feeding level of 0.435 kg/d. In a review of results from 276 castrated lambs offered concentrates at a range from 0.4 to 1.2 kg/d, Keady and Hanrahan (2015) revealed that sheep offered high-quality grass silage had a higher concentrate substitution rate (0.53 vs. 0.31 kg/kg) than those given medium-quality grass silage. The higher concentrate substitution rate in the present study might be due to the high-quality grass used (energy digestibility of 0.80).

It is generally considered that NDF content is the primary chemical component determining intake and digestibility (McDonald, 2011). In this study, NDF concentrations in grass-only diet and concentrate supplementation diet were 0.456 vs. 0.413 kg/kg DM, such a difference might not be large enough to produce a significant difference in total DM intake and digestibility between the two diet treatments. A further factor responsible for no significant increase in feed intake and digestibility for concentrate supplementation in this study might be from the low concentrate input (dietary concentrate proportion was 24.2%). There is evidence indicating that feeding concentrates up to 30% of the total diet with good quality forges at a lower feeding level (< 20 g DM/kg BW) had no significant effects on NDF digestibility (Udén et al., 1984), although the optimum efficiency of ruminal microbial population was improved after concentrate feeding levels reached to 30% to 40% (Archimède et al., 1997). Indeed, in this study, there was no significant difference between the two diet treatments in any variable in digestibility or efficiency of utilization of energy or nitrogen.

### Effects on Nitrogen Utilization Efficiency

Nitrogen intake is the primary predictor for estimating manure N excretion (Yan et al., 2007; Dong et al., 2014), and more than 70% of ingested N exceeding the requirement of microbial synthesis would be excreted in urine (Castillo et al., 2001; Huuskonen et al., 2014). A number of previous studies found that increasing 1 g of N intake could increase N excretion in urine and feces, respectively, by 0.51 and 0.20 g for beef cattle (Dong et al., 2014), 0.51 and 0.38 g for heifers and no lactating cows (Reed et al., 2015), and 0.45 and 0.12 g for sheep offered fresh ryegrass (Zhao et al., 2016b). The corresponding values in the present study were 0.56 and 0.18 g, respectively. Hence, reducing N intake by manipulating dietary N concentration is the most effective strategy to decrease N excretion in manure. For example, decreasing dietary N concentration by 1 g/kg DM could reduce N excretion per kg BW by 0.089 g and urinary N output as a proportion of N intake by 0.76% in beef cattle (Yan et al., 2007). Reducing CP concentration in dairy cow diets to about 0.16 kg/kg DM could reduce ammonia production by 20% (Kebreab et al., 2002). A similar reduction in proportionating N excretion by reducing dietary N concentration was also observed in the studies of Castillo et al. (2001) with dairy cows, Silva et al. (2004) with sheep, and Islam et al. (2000) with goats. However, feeding sheep diets with low CP level at 0.11 kg/kg DM could decrease the efficiency of N utilization (Silva et al., 2004).

The N utilization efficiency can be improved by formulating balanced diets that supply sufficient fermentable energy for rumen microbes to capture ammonia for protein synthesis (Dijkstra et al., 2011). It was observed that the concentrate supplementation was an effective way to improve microbial protein synthesis and shift N excretion from urine to feces (Islam et al., 2000; Zhao et al., 2015). However, in the present study, feeding concentrates at 0.5 kg/d had no significant effects on N utilization efficiency in terms of N excretion or retained N as a proportion of N intake. This might be attributed to the high-quality fresh grass (energy digestibility = 0.80) used in this study, which contained 0.150 and 0.215 kg/kg DM of CP and WSC, respectively. The disagreements could be mainly contributed by the differences in the nature and quality of concentrates and forage that determine the supply of energy and N to the rumen microbe and host animals (Fanchone et al., 2013). The synchronous supply of N and fermentable energy to the rumen is essential to maximize the microbial growth and consequently N utilization efficiency (AFRC, 1993).

### Effects on Enteric CH_4_ Emission

Feed intake is the primary driver for enteric CH_4_ emissions, which contributes 81% of the variation in daily CH_4_ production measured in chambers with fresh ryegrass-based diets (Hammond, 2011), and daily CH_4_ emission (MJ/d) is expected to increase by 20% for a 10% increase of dietary NDF concentration (Ellis et al., 2007). In the present study, the diet added 0.5 kg/d concentrates slightly increased total DM intake, but decreased dietary NDF concentration by 4.29%, consequently the diet treatments had no significant effects on CH_4_ production, although total CH_4_ emission (g/d) was marginally higher with sheep offered concentrates.

Increasing concentrate inputs normally reduces the dietary NDF concentration while increasing ME concentration, thus reducing CH_4_ emissions per kg DM intake in cattle (Ellis et al., 2007; Yan et al., 2010). This reduction was observed in previous studies with goats offered Italian ryegrass pellet supplemented with 50% of corn (Islam et al., 2000), with growing cattle in a regression study (Yan et al., 2009), and with dairy cows in a literature review of published data (Ellis et al., 2007). The forage quality plays a very important role in determining the extent to CH_4_ production. The CH_4_ emission as a proportion of DM and GE intake was similar between yearling steers grazed on pasture only and supplemented with barley, but these variables were significantly affected by grazing season (Boadi et al., 2002). The CH_4_ emission per kg DM intake for ewe lambs fed intensively managed ryegrass was lower than that fed extensively managed permanent pasture (Fraser et al., 2015). In the current study, supplementation of concentrate at 0.5 kg/d to sheep offered very good quality grass had no effects on CH_4_ emission as a proportion of DM intake or GE intake.

The CH_4_ production per unit of intake is negatively related to the level of feeding, because increasing feed intake can speed up the outflow rate of rumen digesta and leave less time for rumen microbial fermentation (Eng et al., 1964; Silva et al., 2004). For each increase in multiple of intake levels above ME requirement for maintenance, the percentage of GE intake loss as CH_4_ decreased by an average of 1.6% for sheep fed concentrate diets (Johnson and Johnson, 1995) and fed ryegrass (Hammond, 2011); 1.2% for sheep offered fresh, ensiled, and pelleted ryegrass (Zhao et al., 2016a); and 0.91% for lactating dairy cows offered diets based on fresh grass or grass silage (Yan et al., 2010). In the current study, because the supplementation of concentrates by 0.5 kg/d had little effects to nutrient digestibility or levels of feeding, there were no dietary effects on CH_4_ emission as a proportion of DM intake or GE intake.

In this study, CH_4_ energy loss as a proportion of GE intake was 0.050 or 0.053 for diet without or with concentrates supplement. These values are much lower than that the value (0.065) recommended by the Intergovernmental Panel on Climate Change (IPCC, 2006) for calculation of enteric CH_4_ emissions for sheep where the measurement data are not available. The present results indicate that using the recommendation of IPCC (2006) might cause a certain level of error for prediction of enteric CH_4_ emissions from sheep grazing on good quality grass pasture. Further studies are required to quantify enteric CH4 emissions of sheep grazing on various qualities of grass pasture.

## CONCLUSION

This study showed that neither concentrate supplementation nor ewe breed had significant effects on nutrient digestibility, efficiency of utilization of energy or N, or enteric CH_4_ emissions when sheep were offered good quality of zero-grazed grass. These results indicate that sheep can utilize good quality grass as effectively as that including concentrates in the diet. Thus, improving grazing grass quality is the key to improve the nutrient utilization efficiency and mitigate N excretion of sheep production.

*Conflict of interest statement*. None declared.
