# Erratum: Effects of concentrate input on nutrient utilization and methane emissions of two breeds of ewe lambs fed fresh ryegrass

**DOI:** 10.1093/tas/txy128

**Published:** 2018-11-30

**Authors:** Chunmei Wang, Yiguang Zhao, Aurélie Aubry, Gareth Arnott, Fujiang Hou, Tianhai Yan

**Affiliations:** 1 Agri-Food and Biosciences Institute, Hillsborough, UK; 2 State Key Laboratory of Grassland Agro-Ecosystems, Key Laboratory of Grassland Livestock Industry Innovation, Ministry of Agriculture and Rural Affairs, College of Pastoral Agriculture Science and Technology, Lanzhou University, Lanzhou, Gansu, China; 3 Institute for Global Food Security, School of Biological Science, Queen’s University Belfast, Belfast, UK

In “Effects of concentrate input on nutrient utilization and methane emissions of two breeds of ewe lambs fed fresh ryegrass” by Wang et al [https://doi.org/10.1093/tas/txy106], the author “Hujiang Hou” should be “Fujiang Hou”. Additionally, in the sentence: “The concentrate contained 0.878 kg/kg DM, 17.8 MJ/kg DM of GE, and 0.069, 0.206, 0.177, 0.119, and 0.023 kg/ kg DM of ash, CP, NDF, ADF, EE, and GE concentrations, respectively.”, “ADF, EE, and GE” has been changed to “ADF and EE”.

